# Application of root cause analysis and TEAMSTEPPS post intravesical gas explosion during transurethral resection of the prostate: a rare case report

**DOI:** 10.1186/s12894-024-01520-w

**Published:** 2024-07-04

**Authors:** I-Hung Chen, Cher-Min Fong, Hsing-Hua Stella Chang, Ying-Jui Ni, Kon-Ning Chiu, Kai-Wen Lee

**Affiliations:** 1 Department of Internal Medicine, National Defense Medical Center, Taipei City, Taiwan; 2 Institute of Medical Science and Technology, National Sun Yat-sen University, Kaohsiung City, Taiwan; 3 Department of Business Management, National Sun Yat-sen University, Kaohsiung City, Taiwan; 4 International Master of Business Administration, National Taichung University of Education, Taichung City, Taiwan; 5 Department of Medicine, Kaohsiung Armed Forces General Hospital, Kaohsiung City, Taiwan; 6 Department of Urology, Kaohsiung Armed Forces General Hospital, Kaohsiung City, Taiwan; 7 Kaohsiung Armed Forces General Hospital, Kaohsiung City, Taiwan

**Keywords:** Transurethral resection of the prostate, Intravesical gas explosion, Root cause analysis, Team strategies and tools to enhance performance and patient safety

## Abstract

**Background:**

An intravesical gas explosion is a rare complication of transurethral resection of the prostate (TURP). It was first reported in English literature in 1926, and up to 2022 were only forty-one cases. Injury from an intravesical gas explosion, in the most severe cases appearing as extraperitoneal or intraperitoneal bladder rupture needed emergent repair surgery.

**Case presentation:**

We present a case of a 75-year-old man who suffered an intravesical gas explosion during TURP. The patient underwent an emergent exploratory laparotomy for bladder repair and was transferred to the intensive care unit for further observation and treatment. Under the medical team’s care for up to sixty days, the patient recovered smoothly without clinical sequelae.

**Conclusions:**

This case report presents an example of a rare complication of intravesical gas explosion during TURP, utilizing root cause analysis (RCA) to comprehend causal relationships and team strategies and tools to improve performance and patient safety (TeamSTEPPS) method delivers four teamwork skills that can be utilized during surgery and five recommendations to avoid gas explosions during TURP to prevent the recurrence of medical errors. In modern healthcare systems, promoting patient safety is crucial. Once complications appear, RCA and TeamSTEPPS are helpful means to support the healthcare team reflect and improve as a team.

## Background

Transurethral resection of the prostate (TURP) stays the gold standard for the treatment of lower urinary tract obstruction due to benign prostatic hyperplasia [1–3]. The complications during TURP include bleeding, ureteral injury, perforation, etc. [4, 5]. We want to share a rare case of bladder perforation during TURP caused by the intravesical gas explosion. An intravesical gas explosion is a rare complication of TURP which was first reported by Cassuto in 1926, with only about forty-one cases reported so far until 2022 [5–8] with an incidence rate of 0.01–0.02% [9–11]. Most of the reported patients recovered and were discharged, but two unfortunately died [11, 12]. The intravesical gas explosion can result in three kinds of injury: (1) Subclinical explosion. (2) Mild injury. (3) Severe injury [5, 13]. Preventive strategies previously proposed in the literature include minimizing the operation duration, reducing the cutting and coagulation current power, using a continuous irrigation approach, paying attention to eliminating air bubbles during the TURP, and having good interaction between the surgeon and the anesthetist [11, 12, 14]. It is of great significance to report this rare surgical complication, grasp its mechanism, and offer modification and prevention strategies to effectively prevent the recurrence of such medical mistakes.

## Case presentation

A 75-year-old man was admitted due to urine retention for 6 months. He had a past history of benign prostate hyperplasia (BPH) for several years and started Foley insertion in the past 6 months and hypertension. Since the Foley catheter had been inserted for half a year, the patient himself requested surgery. On coming to our ward, he was lucid. His body weight was 84.1 kilograms, body height was 161.6 cm. During the initial evaluation, no aberrations were found in routine laboratory studies or on physical assessment. Echocardiography revealed normal regional wall motion with preserved left ventricular systolic function. We evaluated the size of the prostate, which was 60 grams and the PSA was 2.217 ng/mL. Since patient’ s past medical history has hepatocellular carcinoma post-operation, diabetes, hypertension, cardiac arrhythmia, and bronchial asthma; we recommended patients to utilize TURP and provided surgical types such as Thulep. Finally, the patient and his family chose TURP.

**Fig. 1:**
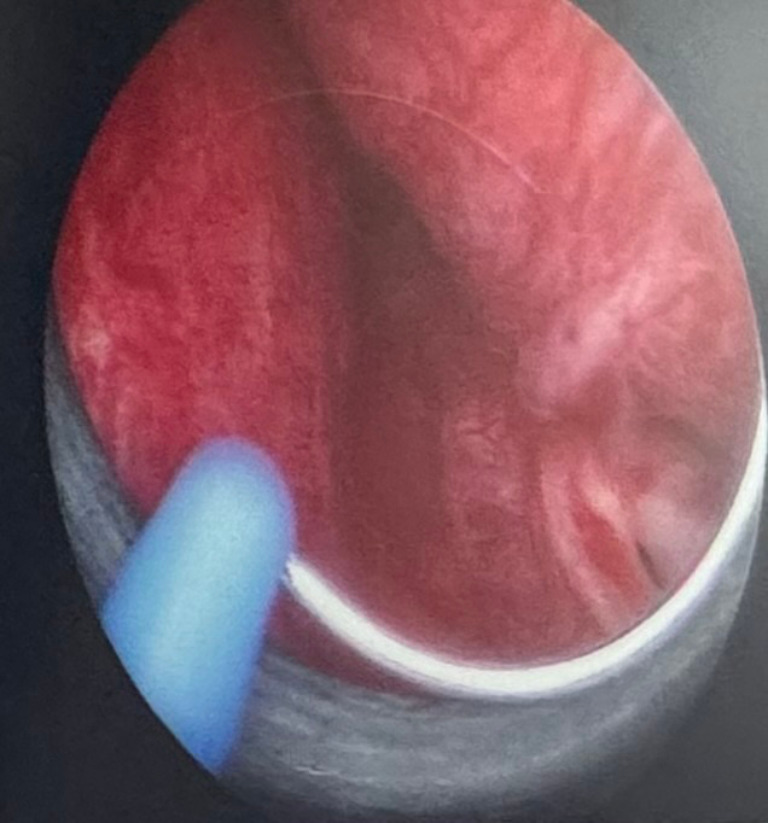
Endoscopic view of tear on the sidewall of the bladder

The patient was prepared to undergo TURP the following afternoon. The bipolar electrocautery current was set at 80 watts for coagulation and 120 watts for cutting. Nearly three hours into the operation, toward the end of the process and during coagulation of some of the bleeding points. A loud explosion was heard while reaching the anterior aspect of the bladder neck at the 12 o’clock position, and a jolt was felt in the lower abdomen. The endoscopic examination of the bladder revealed a tear on the sidewall of the bladder (Fig. 1). After an explanation to the family, we adopted an immediate exploration laparotomy of abdominal organs including the major vessels. Under exploratory laparotomy, there was a large stellate-like perforation in the bladder confirming the intravesical gas explosion occurred with an intraperitoneal bladder perforation.

Since the injury was severe, the patient received emergent exploratory laparotomy with bladder repair surgery and the postoperative medical process of care was not smooth. A total of sixty-five grams of prostate tissues were scraped in this operation, and no malignant cells were found in the pathological examination. Subsequently, the patient was transferred to the intensive care unit.

The patient’s post-operative medical care process was not smooth, and he was also complicated by urinary tract infection and respiratory failure with mechanical ventilator dependence. Fortunately, under intensive care for up to sixty days by the medical team, the patient was successfully weaning the ventilator and discharged without clinical sequelae.

## Discussion and conclusions

TURP was found to be safe in surgery for benign prostatic hyperplasia (BPH) and still means the gold standard for managing benign prostatic hyperplasia [1, 2, 15]. Technical options such as bipolar and laser treatments may further minimize the risks of this procedure [1, 2]. Three kinds of surgery for benign prostatic hyperplasia (BPH), transurethral resection of the prostate (TURP), laser vaporization of the prostate (LVP), and laser enucleation of the prostate (LEP) were found to be safe [15]. The mean age of patients who received TURP increased from 70.6 in 1990 to 74.0 years old in 2010. The mean resection weight of the gland stayed unchanging (22.95 g in 1990, 22.55 g in 2000, and 20.76 g in 2010) [16]. Severe postoperative complications are associated with increased surgery duration and increased morbidity was found in patients with a resection time of more than ninety minutes, gland size of more than forty-five grams, and patient age greater than eighty years old [17, 18]. On multivariate analysis, the surgery of transurethral resection of the prostate (TURP) lasting longer than ninety minutes had higher chances of mortality, sepsis, myocardial infarction, venous thromboembolism, and failure to wean from the mechanical ventilator [15–17]. This surgical complication, although rare, can be avoided by taking precautions [19]. Accurate preoperative judgment of the size of the adenomas for predicting the expected resection weight and duration of the surgery is appreciatively desirable [20].

Facing this intravesical gas explosion, what can help answer three basic questions: what happened, why did it happen, and how can it be prevented from happening again? That is root cause analysis (RCA). RCA is a structured, step-by-step retrospective mistake analysis of an adverse event what occurred, the underlying causes, and what can be done to prevent recurrence. In 1997, the Joint Commission on Accreditation of Healthcare Organizations (JCAHO) began to require hospitals and other healthcare organizations to use the RCA process to investigate sentinel events. RCA focuses on the improvement of the entire system and process, rather than the responsibility of individual execution. The causes of mistakes should be explored based on the principle that mistakes may come from system problems and exceptions caused by a series of mistakes. Based on establishing a safety organization and preventing the recurrence of incidents, a safety barrier is established to prevent the recurrence of abnormalities effectively. RCA is now a familiar tool in hospitals, helping to identify and resolve problems to prevent mistakes from occurring again [21]. A book on RCA, published by Joint Commission Resources, and the Canadian Patient Safety Institute and the VA National Center for Patient Safety (NCPS) has developed a series of steps to follow, the basic 5 steps of an RCA can be summarized as follows [22]:

1. Create an RCA team.
2. Gather information.
3. Brainstorm.
4. Identify root causes.
5. Design and implement the action plan.

### Step 1: create an RCA Team

We arranged a root cause analysis investigation team, led by the Medical Director, who is also a top management team member. The facilitator is a specialist from the medical department who is skilled in RCA operations and is responsible for medical quality. The Urology department supervisor and operating room nursing supervisor were invited because they were individuals familiar with the subject matter of the incident. The director of the medical engineering office who is responsible for the maintenance of medical equipment was invited to participate as a consultant.

### Step 2: gather information

To find out the facts about the incident, the RCA team quickly initiated the root cause analysis and investigation team to execute interviews with the personnel involved while the details were still in-depth in their minds and had not been forgotten. Cooperate with the incident’s clinical medical records, surgical records, anesthesia records, and other written information to gather information to complete the root cause analysis timeline series list within 14 days after the incident.

#### Literature review

During TURP, gas is produced by electrocautery. Since the pyrolysis of prostate tissue and hydrolysis of intracellular water, 30–50% is hydrogen, while oxygen accounts for only 3%, which is not explosive [19, 23–25]. The quantity of the mixed gas formed stands directly proportional to the surgery duration [19, 26]. According to the RCA, due to the sixty-five grams of hyperplastic prostate tissue being scraped, resulting in a surgery duration of up to three hours, the surgical team members, such as anesthetists or nurses, neither proactively alert the surgeon to remind the surgical duration. And also, the device of water irrigation was manual, which would let the atmosphere (up to 21% oxygen) get into the bladder along with the flushing water accidentally and mix with the previously accumulated nonexplosive gas (30–50% hydrogen, 3% oxygen, carbon monoxide, carbon dioxide, and hydrocarbons). Then the mixture of gases is explosive. When the electrode sparks came into contact with the combustible gases accumulated in the bladder, an intravesical gas explosion occurred [27, 28].

### Step 3: brain storming

The medical director handles the conference, constantly asking “what” and “why” from each timeline series point until all possible causes and factors related to the adverse event are considered, and doing their best to explore mistakes, differences, and correct practices and formulate improvement strategies. The proximate causes discussed in this Brain Storming conference are:

1. Verbal communication: lack of surgery time reminder.
2. Decision aid: There is no clear definition for exposing high-risk TURP patients so the medical team can’t understand the increased risk of surgery.
3. Hardware design: the irrigation is an open artificial manual water supply system, and the carries in air when pouring water.
4. Clinical status: The patient’s prostate was evaluated to be larger before surgery.

### Step 4: identify root causes

At this step, it’s time to decide what are the actual root causes, as opposed to proximate causes. A root cause is the most basic factor that, if corrected or eradicated, will diminish the risk or prevent the situation from recurring.

From the consequences of this investigation and literature review, we understand that: due to the large volume of the patient’s prostate gland and the prolonged operation duration, some literature points out that a larger amount of hydrogen and hydrocarbons will be produced in the body during prostate curettage → When adding water manually during normal saline irrigation, air (containing 21% oxygen) to be poured into the bladder → When the attending physician utilized electric cautery to stop bleeding, electrode sparks were induced and then an air explosion happened at the 12 o’clock direction.

It is known from this that the long surgery duration and manual pouring of water were the causes of this medical mistake. (Table 1).

**Table 1:** Causes and derivative incidents after root cause analysis

Cause	Derivative Incident
Longer surgery duration ^#^	Hydrogen & hydrocarbons accumulation
Manual pouring water	Air containing 21% oxygen enters the bladder along with the flushing water

#The surgery took three hours since the rather larger hyperplastic prostate gland

The intravesical gas explosion can result in three kinds of bladder injury: (1) Subclinical explosion: no significant injury in the bladder and no particular therapy is needed. (2) Mild injury: injury to the bladder mucosa, including bleeding, laceration, etc., required therapy intraluminally. (3) Severe injury: appearing extraperitoneal or intraperitoneal bladder rupture needed emergent exploratory laparotomy for bladder repair (Table 2) [5, 13].

**Table 2:** Three kinds of bladder injury post intravesical gas explosion

Bladder Injury	Definition
Subclinical explosion	No significant injury in the bladder and no particular therapy is needed
Mild injury	Injury to the bladder mucosa, including bleeding, laceration, etc., required therapy intraluminally
Severe injury	Appearing extraperitoneal or intraperitoneal bladder rupture needed emergent exploratory laparotomy for suture repair

Shi, B.; Ou, Y.; Cai S. A case report and empirical review of intravesical explosion during transurethral resection of prostate. *Asian Journal of Surgery*.**2022**, 45, 2320-2321

### Step 5: design and implement action Plan

Therefore, how to improve equipment, filter out high-risk patients, and work together as a team to manage and monitor patients’ surgical status and risks are the main improvement action plans in this case.

The report, titled “To Err, is Human,” stated that a significant number of deaths were caused by medical mistakes. The report’s principle is that patient safety depends not only on the healthcare team’s advanced therapeutic care techniques but also on how effectively they are performed [29]. The majority of medical mistakes are attributed to inadequate coordination and communication between healthcare workers and healthcare teams. To manage these deteriorations, there must be modifications not only in the communication approaches among healthcare workers but also in the organizational culture in which healthcare services are delivered [30]. Team strategies and tools to enhance performance and patient safety (TeamSTEPPS) is a team-training intervention that offers assurance in helping the alleviation of medical mistakes [31].

Considering that TURP is a time-pressure surgery, in addition to the surgeon, team members must also monitor the surgery together, so our strategy was introducing the TeamSTEPPS for TURP process modification [30, 32]. TeamSTEPPS has four team core skills, situation monitoring, leadership, communication, and shared mental model (Table 3).

**Table 3:** Teamwork skills and definitions

Teamwork Skills	Definitions
Situation monitoring	Capability to create a shared understanding of the team circumstances and utilize appropriate task strategies to survey teammate performance accurately
Leadership	Capability to direct/coordinate team members, evaluate team performance, assign tasks, encourage associates, plan/manage and sustain a favorable team environment
Communication	Initiation of a notice by the sender, the receipt and acknowledgment of the notice by the receiver, and the verification of the notice by the initial sender
Shared mental models	Organizing a knowledge network of the relationships between the assignment the team is performing and how the team members will interact

We apply these four teamwork skills to patient assessments, and equipment updates, and establish intraoperative checkpoints for TURP. We clearly define what the surgeon, anesthetist, and nurse should do to allow team members to actively assess the patient’s condition and remain attentive at all times. These action plans below can modify the process of TURP to improve team performance, attitudes, and knowledge and concentrate on patient safety comprehensively.

1. Patient assessment:

The surgeon announced to the team members the precautions for high-risk patients [33].

(Ex. surgery duration would exceed 90 min) (apply teamwork skills of leadership, shared mental model, and communication).

2. Device update:

Inform team members that flush water is changed to a closed system. (Apply teamwork skills of the shared mental model).

3. Intraoperative checkpoint establishment:

- When the operation time exceeds 90 min, the anesthetist verbally reminds all team members to be alerted (including the surgeon). (Apply teamwork skills of situation monitoring, and communication).
- The nurse then arranges an alarm clock to warn the team every 30 min. (Apply teamwork skills of situation monitoring, and communication).
- When air bubbles are discovered, the surgeon should confirm it verbally to all the team members. (Apply teamwork skills of leadership, situation monitoring, shared mental model, and communication) (Table 4).

**Table 4:** Applied teamwork skills to modify the TURP process

Items of Modification	Applied Teamwork Skills
1.Patient assessment	Leadership, Shared mental model, and Communication
2.Device update	Shared mental model
3.Intraoperative checkpoint	
a. Takes more than 90 minutes	Situation monitoring and Communication
b. Then every 30 min	Situation monitoring and Communication
c. Discover air bubbles	Leadership, Situation monitoring, Shared mental model, and Communication

These trainable teamwork skills include leadership, situation monitoring, shared mental models, and communication, and the outcomes of teamwork skill mastery are performance, knowledge, and attitudes [30].

Root cause analysis (RCA) is the method and tools that can help to identify unfavorable events, near misses, and sentinel events through retrospective and structured analysis and resolve problems by procedural modifications, guideline reinforcement, and training to prevent errors from occurring again has been taken up in healthcare systems, such as Australia, the US, and the UK [20, 34, 35]. Root cause analysis (RCA) concentrates on system vulnerabilities that contribute to the possibility of mistakes, rather than unique ones. To understand the mechanisms contributing to patient harm, the application of strategies that rotate around the patient, the surgeon, and the surgical procedure is supported. Lately, the collection of data from single-case RCAs (Aggregate RCA) was offered to improve understanding of system functioning and to refine the prioritization of interventions that would prevent similar events [36].

TeamSTEPPS carries the benefit of the most developed evidence-based team training and existed designed to manage the cultural problems encountered in the healthcare system, since people who work together in intensive care units, operating rooms, and emergency rooms, often from different disciplines and academic programs [30]. There is a significant of communication and coordination in the delivery of medical care [37]. Strategies in the team training of these healthcare workers are to support a culture of safety, highlight patient safety and promote healthcare as a high-reliability organization [29]. There is a review that is consistent with marked improvement in communication, a decrease in clinical mistakes rates, and improvement in patient satisfaction after the implementation of improvement strategies such as Team Strategies and Tools to Enhance Performance and Patient Safety (TeamSTEPPS), a validated toolkit from the Agency for Healthcare Research and Quality to maintain a sustainable healthcare environment for all stakeholders [38]. Among the recommendations we proposed, good interaction between the surgeon and the anesthetist during the operation can be maintained by utilizing TeamSTEPPS. There is literature assessing the influence of TeamSTEPPS on operating room efficiency and patient safety. TeamSTEPPS includes operating room staff attending briefings to discuss each case of the day, on-time start rates, and postoperative briefings on identified patient safety issues. Using TeamSTEPPS, average case time decreased by 12.7 min (*P* < .001); first start on time improved by 21% (*P* < .001); patient safety issues dropped from 16% initially to 6% at mid-year and remained stable (*P* < .001). TeamSTEPPS is associated with improved operating room efficiency and reduced operating room patient safety issues [39].

In order to avoid surgical complications, strict and practical use of preventive measures, good interaction between surgeon, anesthetist, and nurses, and close monitoring of patient vital signs and surgical processes are required in order to identify the risk of complications early and carry out crucial interventions immediately [26].

We recommend a closed irrigation device to effectively reduce the chance of atmospheric air entering the bladder. Adjust the power of the electric cautery to a moderate level, and the operation duration should be less than ninety minutes as much as possible. To keep air bubbles away from the dome of the bladder, manual suprapubic pressure or changing the patient’s position to Trendelenburg is sometimes effective [19, 28]. Leaning the resectoscope ventrally while emptying the bladder and using Ellik`s evacuator which is easily handled [13, 19, 26, 27, 34, 35, 40]. Suppose air bubbles are discovered during electrocautery (especially when the dome of the bladder is reached). There should be a good interaction between the surgeon and the anesthesiologist during the operation. In that case, they should be sucked out as soon as possible, and the operation should be suspended at any time [11, 19, 23, 24, 37] (Table 5). Among our six recommendations, the items first and second are related to the medical equipment in the hospital. After discussion, they can be included in the standard operating procedure of surgery. The subsequent four items are related to team coordination, cooperation, and interaction, and have different performances depending on each patient (such as operation duration). Therefore, before the operation, the surgeon presides over a brief and quick task reminder briefing so that all medical staff present, including the anesthetist, can fully understand and fulfill their duties and remind each other (such as the patient’s lying position, whether there are air bubbles that need to be sucked) for ensuring the patient safety.

**Table 5:** Recommendations for preventing gas explosions during TURP

Items of Recommendations
1. Closed bladder irrigation device
2. Moderate level of the electric cautery power
3. Operation duration should be less than 90 min
4. The position of Trendelenburg may keep air bubbles away from the dome
5. Stop operation and soon suck out the discovered dome`s air bubbles with Ellik`s evacuator
6. Good interaction between surgeon and anesthetist during surgery

In medicine, some mistakes are preventable [21]. Once complications occur, you should face them honestly, analyze them deeply, find out the root cause of the problem, and work hard to solve it to prevent it from happening again. RCA and TeamSTEPPS are great tools that can help the medical care team in the hospital to reflect and refine the team.

Ethical issues deriving from the early detection of risk factors in health care are often interrelated and complex. A comprehensive ethical analysis is needed to better embed it in normative frameworks and to assess and weigh the expected benefits of early risk factor detection. Timely ethical reflection may help shape responsible and equitable health policies [41].

When rare medical complications occur, maintaining patient anonymity is paramount in the various meetings that lead to a series of reviews, root cause analysis, and the development of new policies and regulations (such as utilizing the TeamSTEPPS method). The second thing to note is the priority of privacy and ethics for physicians and medical staff. We all want to know what happened after the incident occurred to determine whether some systemic defects or deficiencies ought to be improved. This is business-specific and not individual. Once the individual involved regards that this series of self-examinations is all about being blamed and feeling sad, all the measures will be in vain. This will not help future patient care and medical practice.

We made a case report on this rare surgical complication. When the case happened, we utilized the RCA method step by step to outline the causes and develop recommendations. We also utilized the TeamSTEPPS method to pinpoint inappropriate processes and improve them. The recommendations are based on a specific case, further research and evidence are needed to validate the universal applicability. We have caught from the literature that RCA and TeamSTEPPS are statistically significantly helpful for patient safety. After that, we should expand the methods of RCA and TeamSTEPPS to various clinical departments and design prospective controlled studies. We hope to execute digital transformation in actual medical operations as soon as possible so that artificial intelligence (A.I.) can assist in many aspects and have a fool-proof mechanism, making medical mistakes impossible.

## Data Availability

Data are available from the corresponding author on reasonable request.

## Abbreviations

TURP: transurethral resection of the prostate
BPH: benign prostate hyperplasia
TURBT: transurethral resection of bladder tumor
RCA: root cause analysis
TEAMSTEPPS: team strategies and tools to enhance performance and patient safety
US: United States
UK: United Kingdom
